# Multimodal Behavioral Sensors for Lie Detection: Integrating Visual, Auditory, and Generative Reasoning Cues

**DOI:** 10.3390/s25196086

**Published:** 2025-10-02

**Authors:** Daniel Grabowski, Kamila Łuczaj, Khalid Saeed

**Affiliations:** 1 Faculty of Computer Science, Białystok University of Technology, 15–351 Białystok, Poland; kamila.luczaj.109503@student.pb.edu.pl (K.Ł.); k.saeed@pb.edu.pl (K.S.); 2 Department of Computer Science and Electronics, Universidad de La Costa-CUC, Barranquilla 080002, Colombia

**Keywords:** lie detection, multimodal AI, deception analysis, behavioral sensors, machine learning, transformers

## Abstract

**Highlights:**

**What are the main findings?**

- A multimodal deception detection framework combining visual, audio, and language-based reasoning achieved high accuracy on a DOLOS dataset.
- The ViViT-based visual model reached 74.4% accuracy, while HuBERT audio classification showed strong performance on prosodic cues.

**What is the implication of the main finding?**

- Multimodal fusion enhances robustness and interpretability in behavioral biometrics for deception analysis.
- Language-guided models like GPT-5 prompt-level fusion provide explainable AI outputs, facilitating trust and real-world applicability.

**Abstract:**

Advances in multimodal artificial intelligence enable new sensor-inspired approaches to lie detection by combining behavioral perception with generative reasoning. This study presents a deception detection framework that integrates deep video and audio processing with large language models guided by chain-of-thought (CoT) prompting. We interpret neural architectures such as ViViT (for video) and HuBERT (for speech) as digital behavioral sensors that extract implicit emotional and cognitive cues, including micro-expressions, vocal stress, and timing irregularities. We further incorporate a GPT-5-based prompt-level fusion approach for video–language–emotion alignment and zero-shot inference. This method jointly processes visual frames, textual transcripts, and emotion recognition outputs, enabling the system to generate interpretable deception hypotheses without any task-specific fine-tuning. Facial expressions are treated as high-resolution affective signals captured via visual sensors, while audio encodes prosodic markers of stress. Our experimental setup is based on the DOLOS dataset, which provides high-quality multimodal recordings of deceptive and truthful behavior. We also evaluate a continual learning setup that transfers emotional understanding to deception classification. Results indicate that multimodal fusion and CoT-based reasoning increase classification accuracy and interpretability. The proposed system bridges the gap between raw behavioral data and semantic inference, laying a foundation for AI-driven lie detection with interpretable sensor analogues.

## 1. Introduction

Detecting deception remains a persistent challenge across domains, such as forensic analysis, psychology, and artificial intelligence. Traditional lie detection methods, including polygraph tests and physiological sensors, often suffer from limited reliability due to susceptibility to stress, emotional variability, or intentional countermeasures. As a result, researchers have increasingly turned to behavioral indicators such as facial expressions, voice fluctuations, and linguistic patterns as non-invasive signals of deceptive behavior [1].

Recent advances in multimodal artificial intelligence (AI) and large language models (LLMs) have opened new avenues for developing sensor-inspired lie detection systems. Vision transformers, such as ViViT, and audio-based models, like HuBERT, act as high-level behavioral sensors, extracting implicit emotional cues from facial expressions, gestures, and prosodic features. These digital sensory modules can detect micro-expressions, vocal stress, or timing anomalies, traits often associated with deceptive intent.

In parallel, the emergence of multimodal generative models such as GPT-5 prompt-level fusion and GPT-4 has enabled the interpretation of complex behavioral patterns through natural language reasoning. Specifically, chain-of-thought (CoT) prompting allows these models to articulate step-by-step judgments about deceptive behavior, increasing system transparency and interpretability. This fusion of perceptual input and semantic inference reflects a shift toward explainable AI in behavioral analysis.

In this study, we propose a multimodal deception detection framework that integrates behavioral signals from visual and auditory modalities with CoT-enabled language reasoning. Our system leverages pretrained models to simulate perceptual sensing, while using prompting strategies to generate interpretable justifications for predictions. We validate our approach on the DOLOS dataset, which contains high-quality video segments with labeled deceptive and truthful speech. Additionally, we explore a continual learning setup, where emotion recognition serves as a pretext task for deception classification, testing the hypothesis that emotional cues can enhance model transferability.

The goal of this research is not only to improve deception detection performance but also to demonstrate how AI models can emulate sensor functions like observing, analyzing, and explaining, within a unified framework. By combining signal-level feature extraction with language-based reasoning, our approach represents a novel direction in intelligent lie detection systems.

## 2. State-of-the-Art

### 2.1. Traditional Approaches to Lie Detection

The detection of deception has long been a subject of interest in psychology and forensic science. The most well-known method, the polygraph test, is based on measuring physiological responses such as heart rate, respiration, and electrodermal activity while an individual responds to questions. One of the most widely used protocols is the comparison question test (CQT), which compares physiological reactions to control and relevant questions to infer deception. However, polygraph tests have been criticized for high false positives, susceptibility to countermeasures, and lack of a strong theoretical foundation.

To overcome these limitations, alternative techniques such as the guilty knowledge test (GKT) and cognitive load theory have been explored. GKT assumes that deceivers exhibit differential responses when exposed to crime-relevant stimuli. Cognitive load-based techniques measure the effort required to maintain a lie, often through linguistic or behavioral analysis.

### 2.2. Micro-Expression Analysis in Deception Detection

Micro-expressions, brief and involuntary facial expressions, have been extensively studied as indicators of deception. Ekman et al. [2] proposed the facial action coding system (FACS) [3], which identifies subtle facial muscle movements linked to emotional suppression. Research has shown that liars often attempt to control their facial expressions, but micro-expressions escape conscious suppression, making them useful for deception detection [4,5,6].

Recent approaches leverage computer vision and deep learning to automatically detect micro-expressions. The hierarchical transformer network (HTNet) [7] has been proposed to enhance micro-expression recognition by segmenting the face into localized regions (e.g., the eyes and lips) and applying attention-based feature extraction. Other methods, such as local binary patterns (LBP) and optical flow analysis, have also been used to identify subtle facial movements indicative of deception.

Despite advancements, existing micro-expression datasets are often small and lack diversity, making generalization to real-world deception detection challenging.

### 2.3. Multimodal Deception Detection Systems

Multimodal approaches integrate facial expressions, voice analysis, and physiological signals to improve lie detection performance. Previous systems have combined audio–visual embeddings using feature fusion or attention mechanisms. Models such as OpenFace (facial behavior) and eGeMAPS (prosodic features), as well as biometric sensors, have shown improvements in specific contexts; but, they suffer from limited interpretability and a high reliance on handcrafted features or expensive sensor setups.

Recent studies have introduced deep learning frameworks that combine speech (e.g., HuBERT) and video (e.g., ViViT or VideoMAE) to extract affective and behavioral embeddings. These models act as sensor analogues, capturing subtle deception cues without invasive instrumentation. However, their outputs often lack semantic interpretability, raising concerns about their use in sensitive or forensic scenarios [8].

### 2.4. CoT-Based Generative Analysis of Deception

Recent developments in multimodal large language models (MLLMs) have opened up new possibilities for interpreting human behavior in a more descriptive and transparent way. Instead of relying solely on discrete emotion labels or classification outputs, models like AffectGPT [9] demonstrate that deep learning systems can now generate natural-language explanations of emotional states, grounded in visual, auditory, and contextual data.

This shift from categorical prediction to generative reasoning represents a major step forward in human-centered AI. The AffectGPT framework introduces a large-scale dataset (MER-Caption) with detailed descriptions of affective behavior, and pairs it with a model capable of processing video, audio, and text jointly. These models are not only able to detect emotions such as anxiety, hesitation, or nervousness, which are closely linked to deceptive behavior, but also explain what they see and hear, using language that humans can understand.

In the context of deception detection, this descriptive capacity is especially valuable. Micro-expressions, tone of voice, and timing patterns often play a subtle but critical role in identifying dishonest behavior. Traditional classifiers may detect such patterns, but without interpretability, their outputs remain opaque. Multimodal LLMs, by contrast, can act as behavioral sensors that both perceive and articulate their observations. When guided by chain-of-thought (CoT) prompting, these models reason step by step, linking observed behaviors to likely emotional or cognitive states, and ultimately to truthful or deceptive intent.

This approach aligns with recent work using models like GPT-5 prompt-level fusion, which can perform zero-shot video-language reasoning. When prompted properly, such systems can describe behavioral cues in real time, for example, noting that a speaker is “avoiding eye contact while answering a direct question” and explaining how this might indicate dishonesty. This form of explanatory AI not only enhances trust in the system but also brings it closer to how humans interpret complex social behavior.

As the field progresses, these generative, multimodal models promise to bridge the gap between low-level perception and high-level reasoning, forming a core component of interpretable and reliable lie detection systems.

### 2.5. Emotion-Driven Cues in Deception Detection

Emotions play an important role in the expression of deceptive behavior. Psychological theories, such as Paul Ekman’s concept of emotional leakage, suggest that individuals who lie often experience internal emotional states like guilt, fear, or cognitive dissonance. These emotions may involuntarily manifest through facial expressions, voice, or body movements, even when the speaker attempts to suppress them [10,11].

One prominent concept is that of micro-expressions, which are rapid and involuntary facial expressions lasting less than half a second. These brief expressions have been associated with concealed emotions and are often studied as potential indicators of deception. While detecting them can be difficult for human observers, deep learning models trained for facial emotion recognition may be capable of identifying such subtle cues.

In our experiments, we investigated the relationship between emotion and deception by applying a continual learning approach with the ViViT transformer model. The model was first trained on emotion classification using facial visual data. In the second phase, it was fine-tuned to classify deceptive versus truthful behavior. This approach was based on the hypothesis that emotional representations could provide meaningful priors for detecting deception-related signals, such as facial stress or inconsistencies in expression.

However, this method did not lead to a significant improvement in deception classification accuracy compared to training the model directly on the deception task. One possible reason is the domain shift between typical emotion datasets and the spontaneous behavior found in the DOLOS dataset, which includes more subtle and context-dependent expressions. Additionally, individuals who lie may deliberately attempt to suppress emotional cues, reducing the effectiveness of emotion-based models.

Despite these limitations, emotion remains a promising intermediate representation in multimodal deception detection. Future models may benefit from using emotion embeddings as auxiliary features or from applying multitask learning strategies to jointly train for both emotion recognition and deception detection objectives.

## 3. System Overview

The proposed system for multimodal deception detection integrates behavioral perception and generative reasoning using three complementary components: a visual encoder, an audio encoder, and a multimodal language model. Each module plays the role of a soft behavioral sensor, extracting and interpreting cues related to emotional state, cognitive load, and potential deception [12].

### 3.1. Visual Processing with ViViT

We use the Vision Transformer ViViT to process temporal sequences of facial and upper-body expressions. This model captures fine-grained motion patterns, such as micro-expressions, eye movement, and head gestures. These visual signals are essential in identifying subtle indicators of stress, discomfort, or concealment. The extracted visual features are passed downstream to inform the reasoning module. In some configurations, ViViT is pretrained on emotion recognition and then fine-tuned for deception detection using a continual learning setup.

### 3.2. Multimodal Prompt-Level Fusion in GPT-5

In the proposed framework, multimodal information is integrated using **prompt-level fusion** within a large multimodal model (GPT-5). Unlike classical late fusion approaches that combine classifier logits, or architectures such as GPT-5 prompt-level fusion, which natively encode both image and text modalities, our method embeds all available modality information into a structured prompt that is processed by GPT-5 in a zero-shot manner.

The pipeline consists of three main stages:

1. **Preprocessing of modalities:**
  - *Video frames*: Sixteen equally spaced frames are extracted from each video using OpenCV, preserving spatial, facial, and bodily cues while ensuring coverage across the entire utterance.
  - *Speech transcription*: Audio is extracted from the video and transcribed using the OpenAI Whisper-1 model, providing a verbatim text representation of the spoken content.
  - *Emotion metadata*: Paralinguistic emotional features are obtained from the audio signal using the SpeechBrain emotion-recognition-wav2vec2-IEMOCAP model. The predicted categorical label (e.g., *angry*, *neutral*, and *happy*) and its confidence score are recorded.
2. **Prompt construction:** Each modality is converted into a prompt component:
  - The 16 frames are attached to the user message as base64-encoded images.
  - The transcript text is inserted verbatim.
  - The detected emotion and its confidence score are provided in natural language.
  Depending on the ablation configuration, one or more modalities are omitted from the prompt to measure their individual impact.
3. **Reasoning and classification:** The system message instructs GPT-5 to act as a deception detection researcher, evaluating behavioral, verbal, and paralinguistic consistency. The model is required to output only a JSON object with three fields: label (“lie” or “truth”), confidence (0.0–1.0), and reasoning (a short explanation of observed cues). Decoding parameters are fixed (temperature = 0.2, top-p = 1.0) to ensure deterministic output.

This approach allows GPT-5 to jointly reason over multiple modalities without explicit feature concatenation or model fine-tuning. In the ablation study, we systematically evaluate unimodal (Video-only and Transcript-only), bimodal (Video + Transcript, Video + Emotion, and Emotion + Transcript), and trimodal (Video + Transcript + Emotion) configurations to quantify each modality’s contribution.

Our approach differs from prior multimodal lie detection pipelines in two important ways. First, instead of simply stacking pretrained encoders with a shallow fusion layer, we employ prompt-level fusion in GPT-5, where the outputs of each modality encoder (video frames, transcript text, and audio-based emotion) are jointly presented to the model in a unified reasoning prompt. This design allows the large language model to perform cross-modal reasoning implicitly, rather than relying solely on pre-computed feature concatenation.

Second, GPT-5 is a newly released large multimodal model and, to the best of our knowledge, this is among the first peer-reviewed studies to evaluate its performance in deception detection. The model is leveraged in a zero-shot setting, without task-specific fine-tuning, making it directly applicable to new domains and datasets without costly retraining. By integrating affective, visual, and lexical cues in a single reasoning step, the system can generate interpretable justifications alongside classification outputs.

### 3.3. DOLOS Dataset

The DOLOS dataset serves as the foundational benchmark for all experiments conducted in this study. It consists of high-quality video samples extracted from the British television show *Would I Lie to You?* (WILTY), in which participants engage in a structured deception game format. Each clip is annotated as either *truthful* or *deceptive*, providing a naturalistic and challenging setting for multimodal lie detection.

Unlike many synthetic or laboratory-collected datasets, DOLOS captures spontaneous human behavior in a semi-controlled yet socially dynamic environment. The clips feature a wide range of speakers, accents, and emotional expressions, recorded in studio-quality conditions with frontal camera views and clear audio. This diversity makes the dataset suitable for evaluating generalization across individuals and content types.

**Figure 1:**
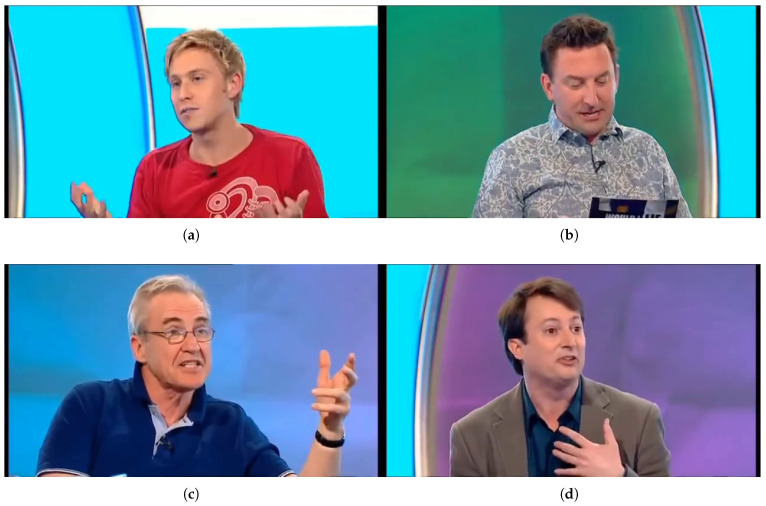
Selected frames from the DOLOS dataset showing representative examples of truthful and deceptive responses. Facial cues and micro-expressions are used in downstream classification. (**a**) Truth sample: participant answering truthfully. (**b**) Truth sample: neutral facial expression. (**c**) Lie sample: subtle signs of discomfort. (**d**) Lie sample: raised eyebrows and eye contact aversion.

Each video sample is accompanied by aligned audio and, in some cases, automatic transcripts, enabling experiments across vision, audio, and language modalities. We selected a subset of DOLOS containing both lies and truths balanced across speakers to ensure fair evaluation. The dataset also provides sufficient temporal depth to analyze fine-grained behavioral cues such as facial micro-expressions, prosodic variations, and gestural inconsistencies. Figure 1 illustrates sample frames from the DOLOS dataset [13].

## 4. Preliminary Experiments

To evaluate the feasibility of multimodal deception detection using transformer-based architectures, we conducted a series of baseline experiments involving visual, auditory, and classical feature-based models. All evaluations were performed on the DOLOS dataset, which contains high-quality audiovisual recordings annotated for truthful and deceptive behavior.

### 4.1. Visual Stream: Video Transformers for Deception Classification

In the visual modality, we utilized ViViT-B/16 and Timesformer-B architectures as spatiotemporal feature extractors. Both models were trained using LoRA [14,15] adapters on short video clips extracted from DOLOS.

**Figure 2:**
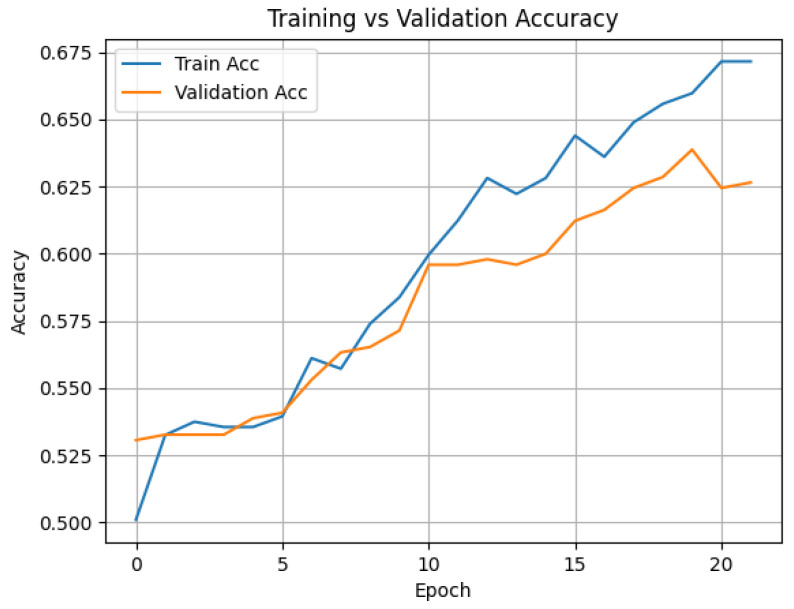
Training vs. validation accuracy for the HuBERT-based audio classification model. Performance improves steadily up to 67.5% with minimal overfitting.

**Figure 3:**
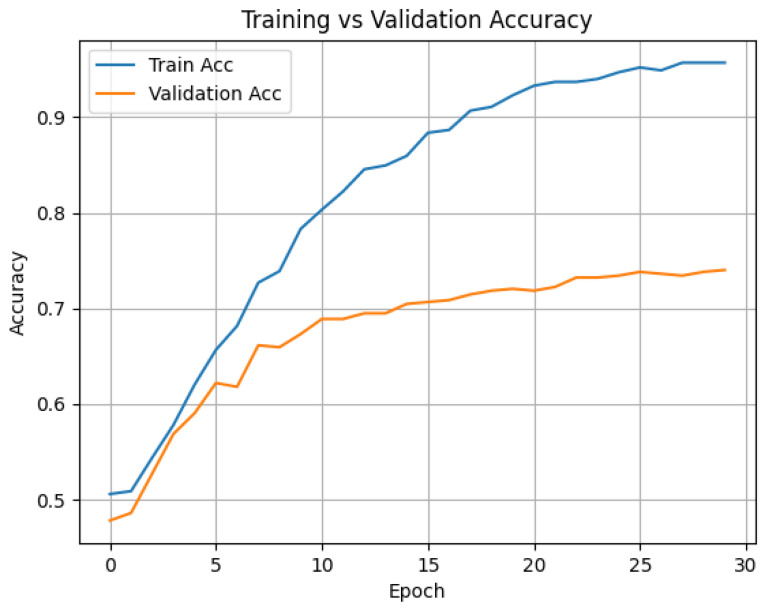
Training vs. validation accuracy for the VideoMAE-based visual classification model.

Two sampling strategies were explored: dense sampling and uniform sampling. The best results were achieved using ViViT-B/16 with uniform sampling and 32-frame clips, reaching an average accuracy of 74.4%. Timesformer-B achieved comparable performance (74.3%) on eight-frame sequences, although required early stopping due to overfitting. Results indicate that visual cues alone provide a solid foundation for deception detection, especially when fine-tuned on domain-specific data. Table 1 presents the performance of ViViT-B/16, Timesformer-B, and ResNet+LSTM models trained on the DOLOS dataset, comparing dense versus uniform sampling along with mean accuracy and key training settings. Figure 2 shows the training and validation accuracy for the HuBERT-based model, and Figure 3 shows the same for the VideoMAE-based model.

### 4.2. Facial Feature Streams: OpenFace and ResNet Backbones

We also experimented with facial feature-based models, using OpenFace landmarks and ResNet embeddings combined with LSTM layers. A frozen ResNet backbone achieved up to 74% accuracy but suffered from significant overfitting, even with regularization techniques such as dropout and weight decay. Fine-tuned ResNet variants did not yield conclusive results [16,17].

### 4.3. Classical Models on Multimodal Features

For comparison, we trained classical machine learning models (SVM, naive Bayes, random forest, and MLP) on MUMIN-style handcrafted features extracted from DOLOS clips. These models achieved accuracy in the 54–58% range, with MLP reaching the highest reported score (58%).

Although these results are notably lower than deep learning-based approaches, they establish a useful lower bound and highlight the complexity of deception classification from static features. Table 2 presents the performance of classical machine learning models on MUMIN features extracted from the DOLOS dataset, including accuracy, F1-score, and brief comments.

### 4.4. Ablation Study

To quantify the contribution of each modality, we perform a systematic ablation study using the GPT-5 prompt-level fusion approach described in Section 3.2. In this setting, all predictions are generated in a zero-shot manner by conditioning GPT-5 on the available modalities embedded within a structured prompt. Ablation is implemented by selectively omitting modalities from the prompt while keeping all preprocessing, prompt formatting, and decoding parameters identical.

We evaluate:

- **Unimodal:** Video frames only (V) and transcript only (T).
- **Bimodal:** Video + Transcript (V + T), Video + Emotion (V + E), and Transcript + Emotion (T + E).
- **Trimodal:** Video + Transcript + Emotion (V + T + E), i.e., the full proposed configuration.

For each configuration, we report the precision, recall, F1-score, accuracy, and Matthews correlation coefficient (MCC) for the binary classification task (lie vs. truth). Confusion matrices are computed using the ground truth labels from the DOLOS dataset. All metrics are macro-averaged to account for class imbalance.

Table 3 summarizes the results. The trimodal configuration (V + T + E) achieves the highest F1-score, indicating that all three modalities contribute complementary information. Bold values denote the best score in each column.

Among the unimodal settings, **video-only** achieves the highest F1-score, suggesting that visual cues alone already encode significant deception-related patterns. Transcript-only performs slightly worse, indicating that verbal content alone may miss nonverbal leakage signals. We do not report an emotion-only configuration, as preliminary trials indicated insufficient stand-alone predictive value. Video cues, while less discriminative in isolation, provide valuable nonverbal signals, such as micro-expressions and gaze aversion. Emotion features derived from audio contribute subtle prosodic patterns that become more informative when combined with other modalities. In particular, adding emotion to the video and transcript improved the F1-score.

In the bimodal setting, the combination of video and emotion (V + E) yields the weakest performance among all tested configurations, with an F1-score of 0.71 and a Matthews correlation coefficient (MCC) of only 0.29. While the recall is exceptionally high (0.97), indicating that the model correctly identifies almost all truthful statements, the precision drops significantly (0.56), leading to a large number of false positives in deception detection. This suggests that emotion cues extracted from audio, when combined with visual frames alone, introduce noise rather than complementary information for the classification task.

Interestingly, when emotion features are incorporated into the trimodal configuration (V + T + E), performance improves notably across all metrics, including a substantial increase in MCC to 0.54 and a balanced F1-score of 0.79. This indicates that emotion features, while insufficient on their own or in the absence of textual context, can provide additional discriminative power when fused with both video and transcript data. Such a finding supports the hypothesis that paralinguistic cues are best interpreted in combination with verbal and visual content rather than in isolation.

Notably, GPT-5 shows a clear tendency to classify true statements correctly (high recall for the truth class) but is less sensitive to lies, a pattern observed across all modality configurations. In the bimodal group, combining visual and textual information (V + T) substantially improves performance over either modality alone, while the inclusion of emotion cues further boosts recall.

These results confirm that GPT-5 is able to leverage multimodal cues effectively when they are presented in a carefully structured prompt. While unimodal setups can provide meaningful predictions, combining modalities yields a consistent improvement in balanced accuracy and F1-score. The observed gains support the hypothesis that visual, verbal, and paralinguistic signals capture complementary aspects of deceptive behavior.

When comparing our zero-shot GPT-5–based system with AffectGPT, we observe competitive performance despite the fact that our approach does not involve any fine-tuning or task-specific training. AffectGPT, which is fully fine-tuned on multimodal sentiment and deception datasets, achieves a mean score of 74.17 across diverse benchmarks, including MER, MELD, IEMOCAP, MOSI, MOSEI, and the SIMS family of datasets. Notably, AffectGPT attains particularly high performance on SIMS v2 (88.99), indicating strong generalization in sentiment-rich conversational contexts. Our trimodal GPT-5 configuration (Video + Transcript + Emotion) attains an accuracy of 75.0% and an F1-score of 0.789 on the DOLOS deception detection benchmark, matching AffectGPT’s mean performance while operating entirely in a zero-shot setting. This result is notable given the absence of gradient-based optimization in our pipeline and the reliance on prompt-level fusion, suggesting that modern large language models can leverage multimodal cues effectively without supervised adaptation.

The DOLOS study by Guo et al. reported, on a full dataset using a three-fold protocol, unimodal video performance of 61.44% ACC, an F1-score of 69.42%, audio performance of 59.19% ACC, and an F1-score of 73.46%. Their best fusion approach (PAVF + multitask) achieved 66.84% ACC and an F1-score of 73.35%. In our experiments on a balanced subset of DOLOS, unimodal video reached **74.4% ACC**, unimodal audio achieved ∼**67.5% ACC**, and prompt-level fusion of video, transcripts, and emotion cues using GPT-5 in a zero-shot setting achieved **75.0% ACC with an F1-score of 78.9%**. While the higher performance of our system is partly attributable to the additional transcript modality and a different evaluation split, it is notable that our zero-shot, prompt-based fusion surpasses the best supervised multimodal fusion results reported by Guo et al. These results highlight the potential of large language models to integrate multimodal cues effectively without task-specific fine-tuning.

### 4.5. Zero-Shot Inference and Testing Procedure

We adopt a **zero-shot inference** strategy, in which GPT-5 is not fine-tuned on the DOLOS dataset. Instead, the model receives modality-specific inputs (video frames, transcript text, and emotion label with confidence) encoded as natural language and images in a single prompt. The prompt template is fixed for all experiments and explicitly instructs the model to output a JSON object containing a predicted label (lie or truth), a confidence score between 0 and 1, and a brief reasoning.

Algorithm 1 outlines the zero-shot, prompt-level multimodal deception detection procedure using GPT-5.
**Algorithm 1** Prompt -level multimodal deception detection (zero-shot GPT-5).**Require:** Dataset D of videos with ground-truth labels y∈{lie,truth}**Require:** Ablation flags: UseVideo, UseTranscript, UseEmotion    1:Initialize metrics containers    2:**for** each sample x∈D **do**    3:    I←⌀,T←⌀,E←⌀    4:    **if** UseVideo **then**    5:        Extract 16 uniformly spaced frames $I=\{f_{0},\ldots ,f_{15}\}$    6:     **end if**    7:     **if** UseTranscript **then**    8:           Extract audio; obtain ASR transcript *T* (Whisper-1)    9:     **end if**  10:    **if** UseEmotion **then**  11:           Extract audio; compute emotion label *e* and confidence $c_{e}$ (SpeechBrain wav2vec2)  12:           E← “Detected emotion: *e* ($c_{e}$)”  13:    **end if**  14:    Build user prompt: include *E* (if any), *T* (if any), instruction to return strictly JSON  15:    Attach frames *I* (if any) as images to the same message  16:    System message: safety + research framing  17:    Query GPT-5 with deterministic decoding  18:    Parse first valid JSON object: {label, confidence, reasoning}  19:    $\hat{y}\leftarrow$label                                                                        ▹ final class: lie or truth  20:    q←confidence∈[0,1]                             ▹ used only for analysis/threshold sweeps  21:    Store $\left(\hat{y},q\right)$ and compare with ground truth *y*  22:**end for**  23:Compute Accuracy, Precision/Recall/F1 per class, Macro-F1, MCC, Cohen’s κ

### 4.6. Computational Environment

All experiments were conducted using high-performance GPU resources (NVIDIA Corporation, Santa Clara, USA). A100 40GB and 80GB cards available on institutional clusters. To accommodate large batch sizes and memory-intensive models such as ViViT [18], we employed DeepSpeed with ZeRO-3 optimization. This setup enabled efficient distributed training and rapid experimentation across multiple modalities. For smaller-scale tasks, RTX-class GPUs were also sufficient, particularly when using lower batch sizes.

### 4.7. Fusion Experiments and Modality Ablations

We also explored multimodal fusion strategies to combine visual and auditory information for enhanced deception detection. Both early fusion (feature-level concatenation) and late fusion (ensemble of modality-specific classifiers) were considered.

Preliminary findings suggest that each modality provides distinct and complementary cues: the visual stream captures facial expressions and microgestures, while the auditory stream encodes paralinguistic features such as hesitations and intonation changes. Ablation studies confirmed that removing either modality leads to a drop in performance, reinforcing the benefit of multimodal integration.

**Figure 4:**
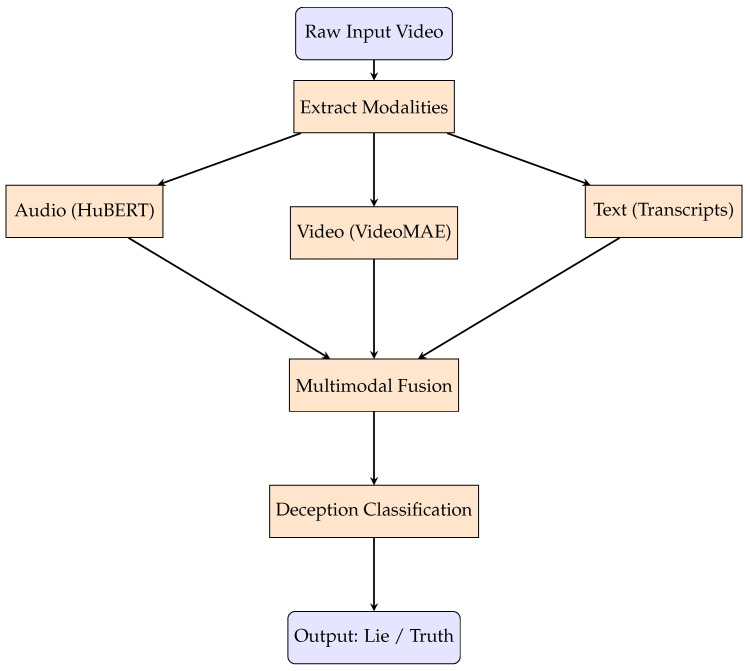
Block diagram of the multimodal deception detection pipeline. Each modality is processed individually before fusion and final classification.

Experiments involving late fusion of HuBERT [19] and VideoMAE [20] models are currently ongoing. While early observations indicate potential synergy between these modalities, full evaluation on the DOLOS dataset has not yet been completed. These fusion pipelines are expected to improve generalization and robustness in real-world scenarios and constitute a promising direction for future research. Algorithm 2 summarizes the end-to-end multimodal deception detection experiment pipeline, from loading DOLOS data to model training, fusion, and evaluation. Figure 4 shows the block diagram of the multimodal deception detection pipeline: audio (HuBERT), video (VideoMAE), and transcripts are processed separately and then fused for final classification.
**Algorithm 2** Multimodal deception detection experiment pipeline.  1:**Input:** DOLOS dataset with audiovisual clips and labels  2:**Output:** Model performance metrics  3:**Load** video and audio samples from DOLOS  4:**for** each sample in dataset **do**  5:    Extract video frames and audio waveform  6:    Generate transcript via speech-to-text model  7:**end for**  8:Split dataset into training, validation, and test sets  9:**Train ViViT model:**  10:   Preprocess video frames  11:   Train transformer on frame sequences  12:   Evaluate on validation set  13:**Train HuBERT model:**  14:   Extract speech embeddings  15:   Train classifier on HuBERT features  16:   Evaluate on validation set  17:**Run GPT-5 prompt-level fusion (zero-shot):**  18:**for** each test sample **do**  19:    Create Chain-of-Thought prompt using transcript and visual cues  20:    Generate textual reasoning and decision  21:**end for**  22:**Fusion:**  23:   Combine ViViT and HuBERT predictions (late fusion)  24:   Evaluate fused model accuracy  25:**return** accuracy and interpretability analysis

## 5. Future Work

The preliminary results demonstrate the feasibility of integrating multimodal deep learning with generative reasoning for deception detection. However, several areas remain open for further exploration and improvement.

One of the key directions involves refining the interaction between perception and reasoning. While current results show promise with zero-shot prompting using GPT-5 prompt-level fusion, more structured prompt engineering and fine-tuning on task-specific reasoning paths could enhance consistency and robustness. In particular, chaining outputs from ViViT and HuBERT directly into CoT sequences via intermediate symbolic representations may offer greater interpretability.

Additionally, the integration of physiological and biometric signals represents a natural extension of the system. Signals such as heart rate variability (HRV), galvanic skin response (GSR), and pupillometry, collected via wearable sensors, can serve as additional behavioral modalities, enriching the model’s perception of stress and deception. This aligns well with the thematic scope of Sensors, where multimodal sensing systems are central.

## 6. Conclusions

This study presents a novel multimodal system for deception detection that integrates visual, auditory, and generative reasoning components. By combining transformer-based models such as ViViT and HuBERT with a large multimodal language model (GPT-5 prompt-level fusion (replacing mPLUG-Owl for our final experiments)) guided by chain-of-thought prompting, we aim to bridge the gap between low-level behavioral signal processing and high-level, human-understandable inference.

Our preliminary experiments on the DOLOS dataset demonstrate that each modality contributes complementary information to the deception detection task. The ViViT model, trained with LoRA on 32-frame segments using uniform sampling, achieved a classification accuracy of 74.4%, marking the highest performance among the tested unimodal approaches. Audio-based classification with HuBERT also showed promising results, especially in detecting paralinguistic cues such as pitch variation and speech hesitation. Although early fusion and late fusion experiments are still ongoing, initial indications suggest that combining modalities may improve robustness and overall performance.

Notably, the generative reasoning approach using GPT-5 prompt-level fusion provided not only classification decisions but also transparent, textual justifications for its predictions. This aligns with growing interest in explainable AI and adds interpretive value beyond raw model outputs.

While the current system remains in a research prototype stage, the integration of descriptive behavioral modeling with multimodal sensing paves the way for future systems capable of real-world deployment in forensic, educational, or security contexts. Future work will focus on finalizing multimodal fusion pipelines, improving temporal modeling of behavior over longer sequences, and incorporating additional sensor modalities such as physiological signals to enhance detection accuracy and cross-domain generalization. We also plan to investigate domain adaptation and symbolic reasoning methods to further strengthen robustness and interpretability.

Overall, the findings underscore the potential of combining transformer-based multimodal perception with language-guided reasoning to build transparent and effective deception detection systems. Our approach illustrates how modern AI techniques can be grounded in psychological theory and applied toward complex behavioral understanding tasks.

## Figures and Tables

**Table 1:** Performance of deep learning models trained on DOLOS (video modality).

Model	Accuracy (Mean)	Training Details
ViViT-B-1 6 × 2-kinetics + LoRA (32 frames), dense sampling	0.740	30 epochs, Adam (0.00005), no overfitting
ViViT-B-1 6 × 2-kinetics + LoRA (32 frames), uniform sampling	0.744	30 epochs, Adam (0.00005), no overfitting
Timesformer-B-kinetics + LoRA (8 frames), uniform sampling	0.743	30 epochs, Adam (0.00005), early stopping used
ResNet (frozen) + LSTM	≈0.74	30 epochs, Adam StepLR (0.0005), overfitting noted

**Table 2:** Performance of classical machine learning models on MUMIN features (DOLOS dataset).

Model	Accuracy	F1-Score	Comments
Linear SVM	0.54	0.52	-
RBF SVM	0.55	0.54	-
Bernoulli Naive Bayes	0.56	0.55	Slightly better than other models
Random Forest	0.55	0.55	-
MLP	0.58	N/A	Best among classical models

**Table 3:** Ablation study results for different modality combinations using GPT-5 prompt-level fusion.

Configuration	Precision	Recall	F1-Score	Accuracy	MCC
*Unimodal*					
Video only (V)	0.639	0.883	0.741	0.692	0.415
Transcript only (T)	0.602	0.933	0.732	0.658	0.379
*Bimodal*					
Video + Transcript (V + T)	0.671	0.917	0.775	0.733	0.502
Video + Emotion (V + E)	0.558	**0.967**	0.707	0.600	0.294
Transcript + Emotion (T + E)	0.598	0.917	0.724	0.650	0.355
*Trimodal*					
Video + Transcript + Emotion (V + T + E)	**0.683**	0.933	**0.789**	**0.750**	**0.537**

## Data Availability

The dataset used in this study is based on the DOLOS corpus, which was constructed from publicly available episodes of the television program *Would I Lie to You?* (BBC). Due to copyright restrictions and the processing involved in dataset construction, the dataset cannot be publicly shared. Researchers interested in replicating or extending this work may contact the authors for further guidance on dataset preparation. No new datasets were generated during this study.
